# Emerging Waste-to-Energy Technologies: Solid Waste Solution or Dead End?

**DOI:** 10.1289/ehp.124-A106

**Published:** 2016-06-01

**Authors:** Nate Seltenrich

**Affiliations:** Nate Seltenrich covers science and the environment from Petaluma, CA. His work has appeared in *High Country News*, *Sierra*, *Yale Environment 360*, *Earth Island Journal*, and other regional and national publications.

Incineration is a dirty word in the United States, at least where trash is involved. We’ve been burning municipal solid waste (MSW) since the 1880s. But the dawning of the environmental movement eight decades later cast new light on the nitrous oxides, dioxins, and other chemicals emitted from as many as 600 mass-burn incinerators nationwide, which meanwhile had also grown in size.^1^ ^,^ ^2^ The ecological merits of resource conservation and recycling became another area of growing interest.

**Figure:**
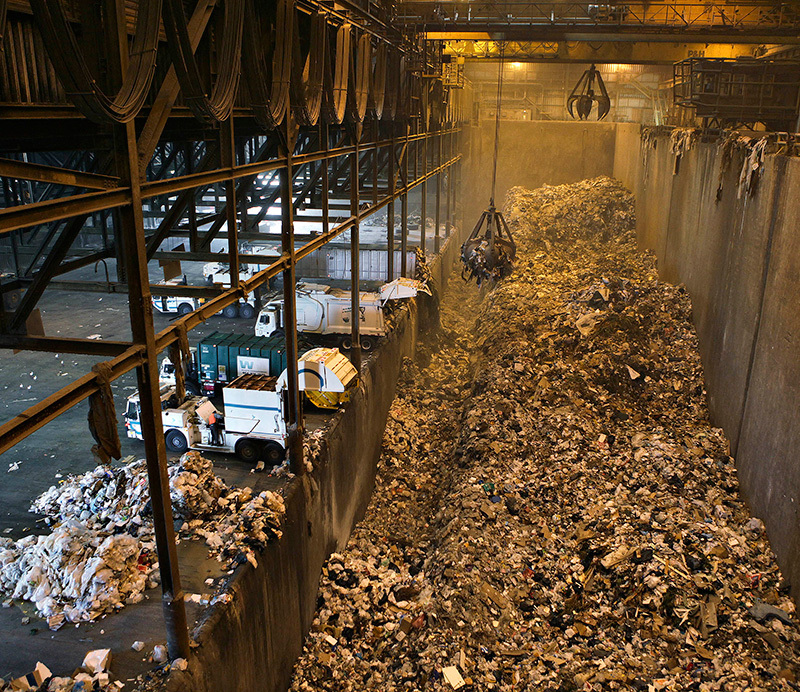
Three emerging thermal waste-to-energy technologies seek to turn municipal solid waste from a burden to an asset. Adherents of these technologies say they produce fewer toxic emissions and virtually eliminate landfilling. But none of the technologies have yet been proven on a commercial scale in the United States. © Bloomberg via Getty Images

Now three new approaches to converting trash into energy—so-called waste-to-energy (WTE) technologies—look to leave mass-burn incineration behind by transforming how we think about MSW in the United States. Adherents of these emerging approaches—gasification, plasma gasification, and pyrolysis—promise cleaner emissions and more flexibility in terms of energy output, plus in some cases the virtual elimination of landfilling through a complex two-stage treatment process.^3^ ^,^ ^4^ ^,^ ^5^ ^,^ ^6^ ^,^ ^7^

But none of the technologies have yet been proven on a commercial scale on U.S. soil using a typical mixed MSW feedstock, says Monica Wilson, program director for the advocacy group Global Alliance for Incinerator Alternatives (GAIA). After years of delays and high-profile failures, the technologies remain stymied by challenges such as operational inexperience, high costs, lack of financing, and concerns about toxic emissions. Furthermore, the heterogeneous nature of MSW can make it a problematic feedstock for power plants, and some critics believe it is more important to assess what materials are actually in MSW and the best uses associated with each of those materials—for instance, recycling, composting, reducing, or redesigning the materials before they enter the waste stream.

Negative public perception of incineration also could prevent acceptance of newer WTE technologies in the United States. Modern mass-burn facilities are a huge improvement over the dirty plants that first drew public outrage. Beginning with the Clean Air Act in 1970, tightened regulations and sophisticated air pollution controls significantly reduced the levels of harmful chemicals emitted by incinerators.

Today, 70 mass-burn plants in 21 states^8^ consume about 13% of the nation’s trash, down from a peak of 14.5% in 1990.^9^ ^,^ ^10^ Cumulatively they offer roughly 2.5 gigawatts of power in return,^11^ ^,^ ^12^ less than a tenth of what the U.S. solar industry produces.^13^ The most recent inventory available from the U.S. Environmental Protection Agency shows that MSW incinerators released about 1% of the quantity of carcinogenic and highly toxic dioxin-like compounds in 2000 that they did just 13 years earlier.^14^

Yet by the 1980s the damage to incineration’s reputation was done, as far as many environmental groups and the public at large were concerned. And the battle lines drawn all those years ago remain largely intact today. So claims that these new technologies offer a panacea to waste management and a source of clean, renewable energy have met with skepticism and organized opposition in dozens of communities nationwide faced with proposals in recent years.^15^

Ultimately, if the new technologies are to take hold in this country, developers must find a way to not only support their performance claims, but also demonstrate compatibility with established recycling and composting efforts and achieve financial feasibility in areas experiencing no shortage of landfill space.^16^ Even some proponents of the new technologies wonder if that will ever happen.

## How Do the Technologies Work?

Those advocates may as well start by getting their facts straight, believes veteran waste-industry consultant and gasification expert Steve Jenkins. Given the technologies’ novelty, developers are prone to misrepresent their projects to the public and to regulatory agencies, he says, often leaving them painted with the same brush as mass-burn incinerators.

Jenkins addressed the issue through a recent presentation to industry representatives reiterating the basic differences between gasification and incineration.^17^ “My purpose was to beat up on the project developers that have not given these technologies and their projects the good credit that they deserve,” Jenkins says. “Too many good projects have died because the public and agency awareness and education were done poorly.”

Gasification, plasma gasification, and pyrolysis are closely related and for the purposes of this article are referred to collectively as “conversion technologies” (the term typically encompasses other noncombustion technologies as well). They involve the super-heating of a feedstock—be it MSW, coal, or agricultural residues—in an oxygen-controlled environment to avoid combustion. The primary differences among them relate to heat source, oxygen level, and temperature, from as low as about 600°F (300°C) for pyrolysis to as high as 20,000°F (11,000°C) for plasma gasification.^18^

In these low-oxygen environments the production of dioxins and furans from waste can be significantly reduced compared with incineration,^19^ ^,^ ^20^ ^,^ ^21^ with emissions potentially falling even below detection limits, Jenkins says. (In one well-publicized exception, a gasification plant in Dumfries, Scotland, repeatedly failed to meet expectations. The plant ultimately closed in 2013 after exceeding emissions limits for dioxins and other pollutants as well as producing far less energy than expected. The Scottish Environment Protection Agency cited “persistent non-compliance with the requirements of the permit” in revoking its license.^22^ ^,^ ^23^)

Conversion technologies are further distinguished from conventional MSW incineration by the production of synthesis gas (or syngas) composed mainly of hydrogen and carbon monoxide, a product of the thermal reactions that take place during the processes. The syngas can then be burned in a boiler system to generate electricity. It can also be processed into fuel for an efficient, low-emissions natural gas generator or refined into other valuable products.^24^

On paper, these differences make conversion technologies cleaner, more efficient, and more valuable than mass-burn incineration. But that doesn’t mean the technologies always perform as advertised. “Engineers from the industry side are evaluating the situation from steady state at maximum temperature,” says Peter Orris, a professor at the University of Illinois who has tracked WTE technologies from a public health perspective. “I don’t have any reason to doubt those estimates are correct, but they’re not necessarily real-world.” That’s because performance is highest and emissions lowest when a facility is running at full steam, he says. Start-up, cool-down, and loading feedstock into the facility’s reactors are when many problems occur.

A gap between potential and actual performance is evident at plants currently operating outside the United States, says Umberto Arena, a researcher based in Italy and associate editor of the journal *Waste Management*. This is due primarily to the lack of an affordable solution for syngas cleaning, he believes.

As it stands today, the cleaning of contaminants and impurities from syngas produced via conversion technologies is often cost-prohibitive.^25^ ^,^ ^26^ ^,^ ^27^ Without an affordable solution for syngas cleaning, which Arena suspects could come in the next five years, most two-stage plants simply burn their syngas in a boiler and then scrub the emissions. This leaves gasification and related technologies only negligibly cleaner than modern mass-burn units and sometimes less efficient.^28^

On top of that, the newer systems must be finely tuned and, in some cases, tend to require a more uniform, pre-sorted feedstock than mass-burn incinerators, Arena says. This can add expense to the operation.

Despite these shortcomings, conversion technologies do have a clear benefit in that they leave behind safer solid residues—and less of it—than burning MSW. Incinerators produce significant amounts of a waste called bottom ash, of which about 40% must be landfilled. The remaining 60% can be further treated to separate metals, which are sold, from inert materials, which are often used as road base. The newer technologies, by contrast, offer immediate recovery of metals and inert slags, with smaller volumes of landfill.^28^ ^,^ ^29^ This explains why conversion technologies have caught on in regions where landfill space is extremely tight or available at a premium.

“If your main interest is to produce electric energy, so far the combustion-based systems are clearly better,” Arena says. “If your interest is to strongly reduce the material that is sent to landfill, as in Japan or Denmark or some other areas of Europe, then you could be very interested in gasification.”

## Landfilling versus Conversion Technologies

Even if the newer conversion technologies have yet to make an unequivocal case that they’re better than their mass-burn predecessors, some argue there’s another comparison that may be more relevant—and ultimately more convincing.

In the United States today, landfills have a big advantage when it comes to economics. Sending trash to the dump is almost always cheaper than burning it, with tipping fees paid by haulers averaging about 33% less at landfills than at existing incinerators, according to one analysis.^30^ That discount would likely be even greater over costly new conversion plants.

But that’s not necessarily a deal-breaker in areas with limited landfill space, such as Los Angeles. “Our focus is to develop an alternative to landfills,” says Coby Skye, a senior civil engineer for the County of Los Angeles Department of Public Works.^31^ The county is running short on landfill space and reluctant to export its trash elsewhere, he says. “We wanted to find something that’s more sustainable.”

In addition to six operating landfills, Los Angeles County already has two mass-burn incinerators and plans to launch multiple commercial-scale anaerobic digesters, which can produce both energy and compost from wet organic wastes.^32^ ^,^ ^33^ “For over a decade, the county has been encouraging these alternatives to kind of wean ourselves off of the reliance on landfill disposal for residual waste,” Skye says.

A study^34^ released in February 2016 by the county Department of Public Works, which Skye helped lead, showed a clear benefit in terms of greenhouse gas emissions for gasification combined with additional recycling and anaerobic digestion, versus the status quo of recycling a portion of MSW and landfilling the rest. The new study compared cumulative greenhouse gas emissions under two different scenarios. The first involved trucking 1,000 tons of post-recycled residuals (i.e., what’s left after current recycling efforts) to a modern dump with a landfill-gas-to-energy system every day for 25 years. This scenario assumed the landfilled residuals would remain there, continuing to break down, for an additional 100 years.

The second scenario involved sending the same 1,000 tons per day of post-recycled residuals to a so-called integrated materials recovery facility. This would include advanced recycling of additional materials followed by processing of residuals via gasification and anaerobic digestion, leaving 136 tons per day for the landfill.

The latter is “what we would call a dream facility,” says Eugene Tseng, an environmental attorney and engineer whose consulting firm helped prepare the report. “You have to have a suite of technologies for what we call the integrated approach. You take the most appropriate technology for the type of waste that’s being generated.”

The study concluded that the landfill scenario would produce a net increase of 1.64 million metric tons of carbon dioxide equivalent emissions over the entire 125 years, while the integrated scenario would result in a net savings of 0.67 million metric tons. This difference of 2.31 million metric tons is comparable to 480,000 fewer passenger vehicles driven for one year. The reduction is achieved in two key ways: 1) through the displacement of emissions from fossil fuel combustion due to the electricity generated; and 2) through increased recycling efforts involved in the extensive pre-processing of materials required before feeding the two plants.^34^

**Figure:**
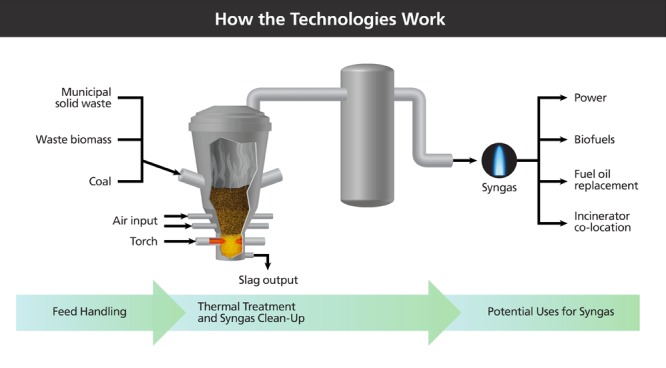
Gasification, plasma gasification, and pyrolysis all involve the super-heating of a feedstock—be it MSW, coal, or agricultural residues—in an oxygen-controlled environment to avoid combustion. The primary differences among them relate to heat source (this example shows torches like those used in plasma gasification), oxygen level, and temperature. Syngas cleaning is necessary for conversion to high-value products such as substitute natural gas but not for combustion in a boiler unit. Illustration: Jane Whitney for EHP

One critical assumption embedded in the study is that biogenic carbon dioxide emissions resulting from the digestion, decomposition, or processing of biologically based materials are considered part of the natural carbon cycle and therefore carbon neutral with zero net greenhouse gas emissions, in accordance with current state, national, and international standards.^35^ ^,^ ^36^ ^,^ ^37^

However, a growing community of scientists and others feel it is inappropriate to consider all of these emissions carbon neutral.^38^ Environmental organizations have called on the U.S. Environmental Protection Agency to account for carbon emitted from biomass waste on the basis that it too can have an immediate impact on climate change, even if it will theoretically one day be reabsorbed by trees and plants.^39^ Such a policy change would mean the landfill scenario in Los Angeles County’s analysis would fare better on greenhouse gas emissions than the state-of-the-art integrated facility.^34^

## Philosophical Differences

The deepest divide between ardent critics and defenders of conversion technologies, and the one that evokes the most passion on both sides, doesn’t have to do with dioxins or energy production or carbon accounting, but rather with philosophies about how to handle our society’s trash—and what, in fact, trash really is.

MSW is a mix of all kinds of materials: not just combustible carbon-based materials but also glass, metals, and more. Proponents of a decades-old philosophy called “zero waste” contend that at least 80% of the typical MSW stream can be recycled or composted (e.g., through anaerobic digestion), and that reuse and waste prevention can reduce the remaining portion—if not all the way to zero, then close.

“The primary ecological benefits associated with recycling are in using recovered materials in a production cycle to displace virgin materials,” says Darby Hoover, a solid waste specialist with the Natural Resources Defense Council (NRDC). “The associated savings of energy, water, and carbon associated with that substitution are where most of the environmental benefits occur. ... That’s the basis of the ‘closed-loop’ idea. Once you introduce a material into commerce, you should do all you can to keep it there.”

Supporters of conversion technologies, meanwhile, contend that recycling and composting aren’t enough to sufficiently improve landfill-diversion rates, and that some sort of thermal processing of leftovers is necessary. They employ the newer term “zero-waste-to-landfill” to allow for conversion technologies and other WTE strategies as an additional and in some cases preferred form of recycling.

“I strongly feel that the goal of zero-waste-to-landfill cannot be achieved without some additional technologies,” says James Stewart, chairman of the California-based industry group BioEnergy Producers Association. His home state has proposed a plan to improve its recycling rate, stuck at around 50% for the last six years,^40^ to 75% by 2020.^41^ (By comparison, nationally an average of 34% of MSW is recycled.^9^) This will require recycling roughly 22 million more tons of the current waste stream, which Stewart considers impractical—unless the state’s plan is rewritten so that “recycling” includes the recovery of energy, carbon, metals, and slag through WTE technologies.

While there’s little overlap between these opposing perspectives, in practical terms it may be possible to find middle ground. For instance, some argue that WTE technologies could be used as a stopgap solution to keep high-value materials out of landfills while traditional recycling efforts continue to ramp up.

**Figure:**
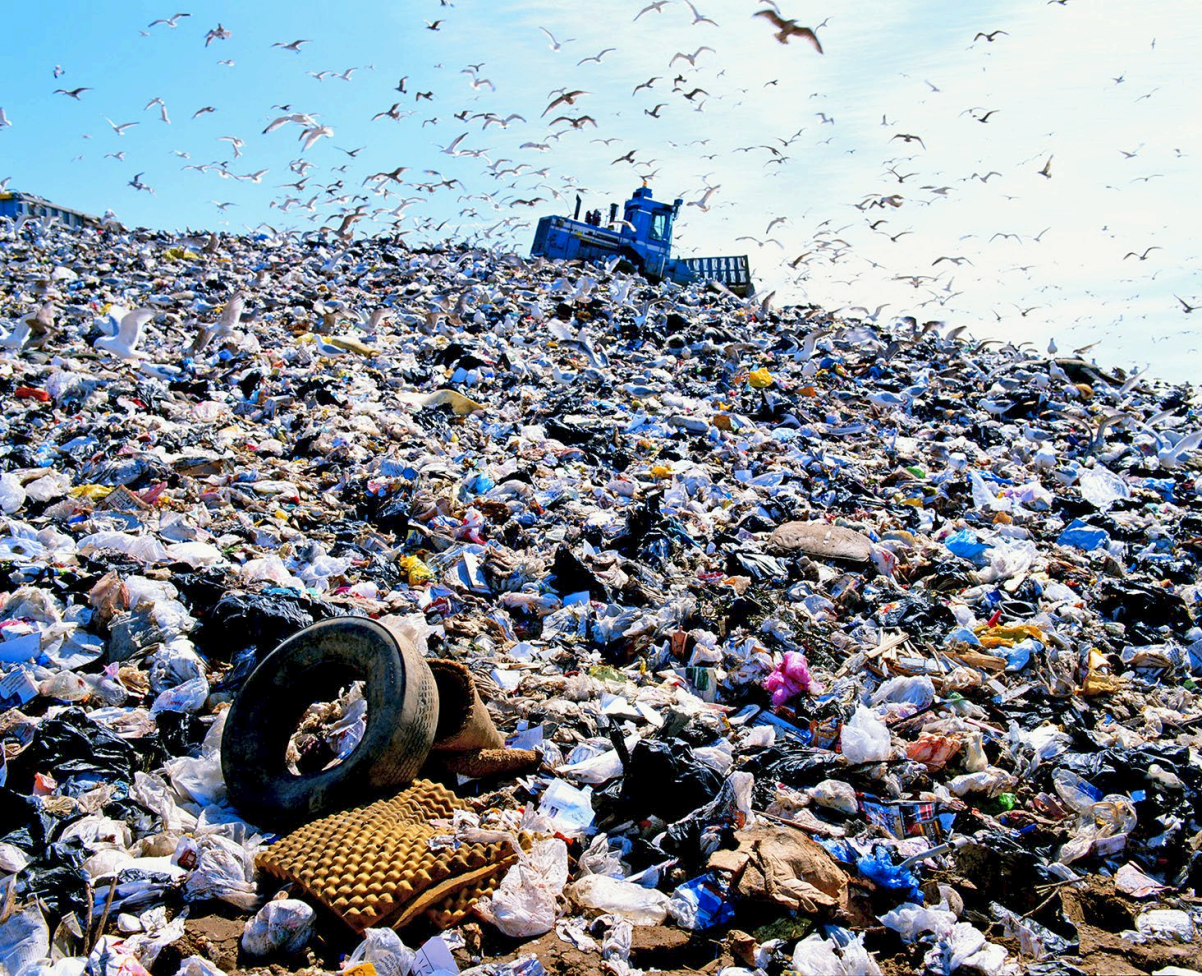
Municipal solid waste comprises a wide variety of materials that most often end up in landfills—nationwide, an average of 34% of the waste stream is recycled. © Stephen Wilkes/Getty Images

**Figure:**
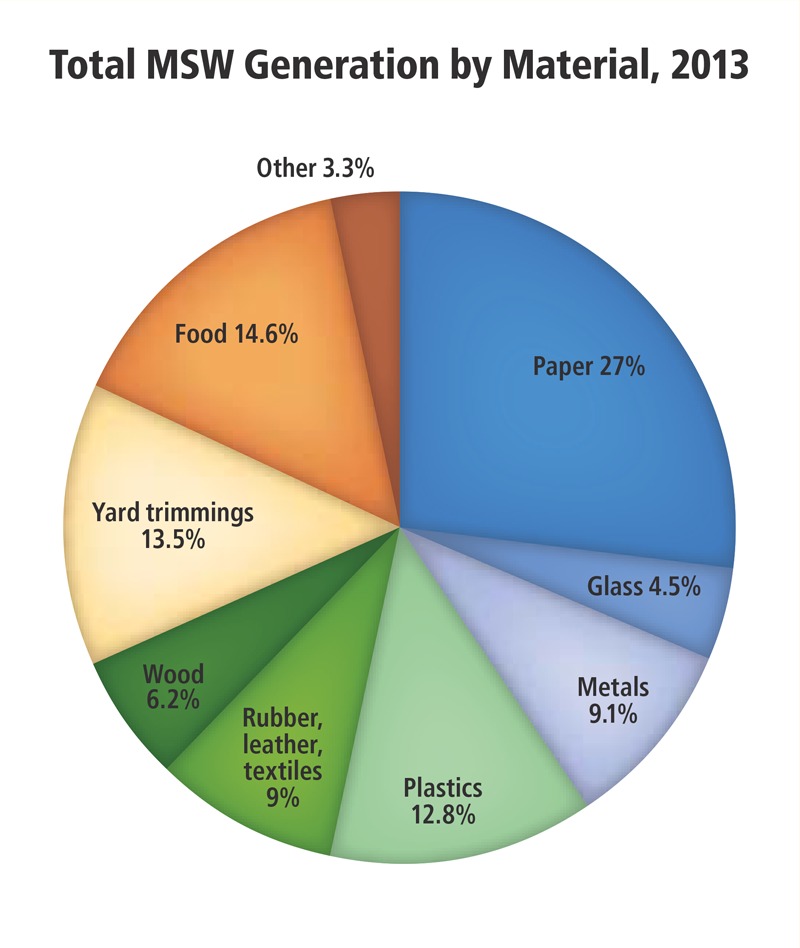
Many of the materials that end up in landfills could potentially produce power in a waste-to-energy plant, although some stakeholders believe recycling and composting should be maximized first. U.S. Environmental Protection Agency

“I really do have sympathy for folks who are saying, ‘It’s a shame that we’re landfilling this stuff, why can’t we take energy out of it until we can recycle more of it?’” Hoover says. “The problem is that interim solutions can interfere with the higher and better ecological pathway. We want to really make sure we’ve actually maximized recycling and composting before we get to the waste-to-energy facilities.” That said, Hoover believes there might be a role for some types of WTE technologies as a last-ditch effort to extract energy from materials that contain it before they go to a landfill.

Shlomo Dowen, coordinator of the advocacy group United Kingdom Without Incineration Network, believes the traditional conception of zero waste remains within reach—and precludes conversion technologies entirely. “There is very little by way of ‘unrecyclable’ material that could not be addressed by redesign, better source separation, and better sorting technologies,” he says. “And much of what would remain would probably have little or no calorific value so would not be suitable for energy recovery.”

## Not Giving Up

Rod Bryden is among those convinced that conversion technologies deserve a place in solid-waste management. The prominent Ottawa businessman runs Plasco Energy Group, a company that hopes to recover power from trash via a proprietary plasma-based technology.

These plans have suffered a number of setbacks, most notably in Plasco’s Canadian hometown early last year.^42^ ^,^ ^43^ ^,^ ^44^ Plasco had attracted $400 million in investments since its formation in 2005^45^ and was considered a leader in the WTE field.^46^ But in February 2015, roughly one year after investors asked Bryden to step down as CEO, Ottawa officials terminated the city’s relationship with Plasco due to its inability to secure additional financing needed to construct a long-awaited new plant for full commercial operations. This plant had been contracted to process about 330 tons of post-recycled garbage per day—roughly a third of the city’s household waste. That same day, the company filed for bankruptcy and laid off 80 employees.^47^

But that wasn’t the end of it. Seven months after Plasco’s collapse, Bryden bought the company from its creditors for a dollar. Now he’s back at the helm of a restructured company planning its second act.^48^ Bryden says that a failure of leadership, not technology, led to the Ottawa plant’s demise, and that Plasco is poised to re-enter the conversion technology industry. “My own view is that Plasco is worth whatever it was created for—$400 million—and has a technology that is ready for commercial delivery,” he says.

Instead of using syngas to generate electricity on site for sale to a power company, the company now intends to produce and sell a cleaner fuel-grade syngas for blending with natural gas in third-party power plants. “We were never an expert in the power business, and we’re not in that business anymore,” Bryden explains.

Tellingly, Plasco’s target market is no longer the United States or Canada, but places like the Chinese capital of Beijing, where Bryden says the price of natural gas is roughly three and a half times what it is in New York. If he can find the right location, he hopes to have a new plant under construction by the middle of next year.

Industry consultant Jenkins remains skeptical about the expansion of conversion technologies in the United States. “[In many areas] we’ve got plenty of land, and nothing is driving the prices of landfills up,” he says. “We don’t have the economic drivers in the U.S. except in a few cities and counties.”

Government subsidies, favorable regulations, and more social acceptance in Japan and parts of Europe have allowed these costly, capital-intensive facilities to advance even as they continue to flounder here. Over the last 40 years Japanese company JFE Engineering has built more than 160 incinerators and 10 gasification plants in its home country, says project development manager Kenny Miyagi.^49^ Hoping to expand to the United States, the company opened a branch in Long Beach, California, in 2012. “Ever since then, we have been chasing after opportunities,” Miyagi says. “It’s a tough market.” Without subsidies, incentives, or other public funding, he says conversion technologies appear unlikely to take off here.

Harvey Gershman, another waste industry consultant familiar with the newer technologies, says he’s been surprised by their continued failure to gain traction in United States. “For the past ten years I’ve been saying ‘within the next four years.’ I was hoping we’d be able to turn the corner on these technologies a lot sooner than we’ve been able to.”

A window of opportunity may already be closing on the technologies, suggests Wilson of GAIA. “There was a flood of proposals, and now there are only a few here and there, and they wither and die on their own. I think the interest overall has really waned over the past year, as people have gotten fed up after hearing so many promises.” But, she concedes, “there still are some really strong believers in it.”

**GLOSSARY**

**Mass-burn incineration**

Mass-burn combustion of MSW occurs in an oxygen-rich setting with minimal prior sorting or preparation. The resulting heat is used to produce steam and electricity.

**Municipal solid waste (MSW)**

MSW is the term for common mixed trash collected from homes, businesses, and institutions, including packaging, food waste, yard waste, and both durable and nondurable goods.

**Synthesis gas (syngas)**

Syngas, composed mainly of hydrogen and carbon monoxide, is produced by conversion technology processes. It can be used as fuel for electricity or converted into other salable products such as liquid fuels.

**Conversion technologies**

This blanket term encompasses noncombustion processes that convert solid waste into useful products. For the purposes of this article, the term refers specifically to gasification, plasma gasification, and pyrolysis, but other conversion technologies include depolymerization, anaerobic digestion, and fermentation.

**Gasification**

Gasification is a process that converts any material containing carbon—such as coal, biomass, or MSW—into syngas. In the controlled presence of oxygen, temperatures of 900–3,000°F (480–1,650°C) break the feedstock molecules apart and recombine them into syngas.

**Plasma gasification**

Plasma gasification uses a plasma torch to provide supplemental heat for the gasification process. Temperatures can reach 5,000–20,000°F (2,760–11,000°C).

**Pyrolysis**

Pyrolysis is a form of gasification that occurs at relatively low temperatures of 600–1,400°F (300–760°C) in the absence of oxygen.

**Waste-to-energy (WTE) technologies**

The full suite of WTE technologies includes thermal processes like mass-burn incineration and gasification as well as nonthermal processes like anaerobic digestion and landfill-gas recovery.

Source: Stringfellow (2014)^18^
